# Testicular Macrophages Produce Progesterone De Novo Promoted by cAMP and Inhibited by M1 Polarization Inducers

**DOI:** 10.3390/biomedicines10020487

**Published:** 2022-02-18

**Authors:** Sawako Yamauchi, Kousuke Yamamoto, Kazushige Ogawa

**Affiliations:** 1 Laboratory of Veterinary Anatomy, College of Life, Environment and Advanced Sciences, Osaka Prefecture University, 1-58 Rinku-Ourai-Kita, Izumisano 598-8531, Osaka, Japan; sawachappy@gmail.com (S.Y.); k.y.930416@gmail.com (K.Y.); 2 Laboratory of Veterinary Anatomy, Graduate School of Life and Environmental Sciences, Osaka Prefecture University, 1-58 Rinku-Ourai-Kita, Izumisano 598-8531, Osaka, Japan

**Keywords:** testicular macrophage, progesterone, mixed culture, Leydig cell, adrenergic receptor

## Abstract

Tissue-resident macrophages (Mø) originating from fetal precursors are maintained via self-renewal under tissue-/organ-specific microenvironments. Herein, we developed a propagation method of testicular tissue-resident Mø in mixed primary culture with interstitial cells composed of Leydig cells from the mouse testis. We examined Mø/monocyte marker expression in propagated testicular Mø using flow cytometry; gene expression involved in testosterone production as well as spermatogenesis in testicular Mø and interstitial cells propagated by mixed culture via RT-PCR; and progesterone (P4) de novo production in propagated testicular Mø treated with cyclic adenosine monophosphate, isoproterenol, and M1 polarization inducers using ELISA. Mø marker expression patterns in the propagated Mø were identical to those in testicular interstitial Mø with a CD206-positive/major histocompatibility complex (MHC) II-negative M2 phenotype. We identified the genes involved in P4 production, transcription factors essential for steroidogenesis, and androgen receptors, and showed that P4 production de novo was upregulated by cyclic adenosine monophosphate and β2-adrenergic stimulation and was downregulated by M1 polarization stimulation in Mø. We also demonstrated the formation of gap junctions between Leydig cells and interstitial Mø. This is the first study to demonstrate de novo P4 production in tissue-resident Mø. Based on previous studies revealing inhibition of testosterone production by P4, we propose that local feedback machinery between Leydig cells and adjacent interstitial Mø regulates testosterone production. The results presented in this study can facilitate future studies on immune-endocrine interactions in gonads that are related to infertility and hormonal disorders.

## 1. Introduction

There are two types of macrophages (Mø) in adults: tissue-resident Mø, which colonize tissues/organs at a steady state and perform tissue-/organ-specific functions to maintain tissue/organ homeostasis, and recruited Mø, which originate from bone marrow-derived blood monocytes that infiltrate lesions in response to tissue/organ damage. Previously, tissue-resident Mø were thought to originate from bone marrow-derived monocytes that undergo tissue-/organ-specific differentiation in adults. However, based on recent evidence derived mainly from mice and partly from humans, most adult tissue-resident Mø have been shown to originate from fetal precursors in the yolk sac and/or fetal liver, which then migrate to different tissues/organs during embryonic development, colonize tissue-/organ-specific microenvironments (niches), and undergo tissue-/organ-specific differentiation into tissue-resident Mø [1].

In mice, and likely in humans, tissue-resident Mø persist into adulthood through continuous proliferation in a steady state, independent of any influx from bone marrow-derived monocytes [1,2,3]. Moreover, tissue-resident Mø are terminally differentiated cells that colonize interstitial tissues of different organs, such as the intestines and dermis, but do not proliferate locally in the colonizing tissues at a steady state. They are known to be gradually replaced by blood monocyte-derived Mø in mice [4,5]. Based on current evidence from mice, Guilliams et al. recently proposed that tissue-resident Mø that receive adequate nourishment under suitable niches can self-renew and undergo tissue-/organ-specific differentiation, eventually gaining tissue-specific functions [6,7]. Therefore, we hypothesized that tissue-resident Mø from fetal precursors in a certain organ can proliferate alongside niche-forming cells residing in the respective organ in vivo. We successfully developed a simple propagation method for tissue-resident Mø using mixed culture with the respective tissue-/organ-residing cells. This method can be applied to mouse tissue-resident Mø from the liver, spleen, lung, and brain [8].

The testis is a male reproductive organ, which plays a crucial role in spermatogenesis and androgen production assigned to the seminiferous tubules and testicular interstitium, respectively. The interstitium is mainly composed of Leydig cells, tissue-resident Mø, vascular endothelial cells, and peritubular myoid cells as cellular components. Testicular Mø express certain immunosuppressive M2 phenotype marker genes and contribute to the establishment and maintenance of immune privilege of the testis, protecting the development of spermatogonia in rodents and humans [9,10]. Two types of tissue-resident Mø reside in the mouse testis. Peritubular Mø, which are located in the layer of peritubular myoid cells surrounding the seminiferous tubules, emerge from bone marrow-derived monocytes after birth. Once settled in the testis, they become long-lived tissue-resident Mø [11]. Peritubular Mø contribute to spermatogenesis by producing factors/cytokines critical for spermatogonial differentiation and regulating testosterone production in mice [12]. In contrast, interstitial Mø residing in the interstitium in close contact with Leydig cells provide a niche for Leydig cells and modulate testosterone production by producing 25-hydroxycholesterol in rats [13,14,15,16]. In mice, these Mø are believed to originate from hematopoietic progenitors in the yolk sac and regulate fetal vascular remodeling and morphogenesis, and they are gradually replaced by bone marrow-derived monocytes in adulthood [11,17]. However, a recent study by Lokka et al. reviewed the origin of testicular Mø in mice [18]. They demonstrated that peritubular Mø are already present at birth and interstitial Mø originate from fetal monocytes differentiated in the fetal liver. Moreover, bone marrow-derived monocytes do not substantially contribute to the maintenance of these two testicular Mø types. Based on a recent study [7], we hypothesized that testicular Mø could be propagated if a suitable niche could be reproduced in vitro. We, therefore, attempted to induce testicular Mø to proliferate in vitro by mixed culture composed largely of Leydig cells putatively providing the niche, using our propagation method of tissue-resident Mø with some modifications. 

Previous studies have revealed that immune cells, including Mø, express diverse steroid hormone metabolic enzymes and steroidogenic acute regulatory protein (StAR), which initiates steroid production, albeit these cells do not express CYP11A1, which is essential for the de novo synthesis of steroid hormones in mammals [19]. Moreover, testicular Mø can produce 25-hydroxycholesterol, which can be used as a substrate for testosterone production in Leydig cells [13,14,15,16]. These previous studies indicated that interstitial Mø may be deeply involved in sex steroid production. Thus, we again hypothesized that testicular interstitial Mø adjacent to Leydig cells can produce sex steroids de novo. In this study, we evaluated this hypothesis by attempting to propagate testicular Mø in mixed culture with interstitial cells substantially composed of Leydig cells, assumed to be niche-forming cells, and further examined the presence of de novo sex steroid production in the propagated Mø. Accordingly, we successfully demonstrated (1) the propagation of testicular Mø presenting identical properties as interstitial Mø in the mixed culture and (2) the de novo production of progesterone (P4) in the propagated testicular Mø, which was upregulated and downregulated by cyclic adenosine monophosphate (cAMP) and M1 polarization inducers, respectively.

## 2. Materials and Methods

### 2.1. Animals

ICR male mice were obtained from Japan SLC Inc. (Hamamatsu, Japan) and housed under specific pathogen-free conditions. In total, we used 32 ICR mice aged 7.5–10 weeks. The animal experimentation protocol was approved by the Animal Research Committee of Osaka Prefecture University (approval number: 19–49, 20–32, 21–26). All experiments were performed following the relevant guidelines of the Osaka Prefecture University. The mice were euthanized by cervical dislocation, and the blood was removed by intracardiac perfusion with Ca-/Mg-free Hanks’ Balanced Salt Solution (HBSS; H6648, Sigma-Aldrich, St. Louis, MO, USA), supplemented with 50 U/mL heparin (224122485, Mochida Pharmaceutical, Tokyo, Japan) when necessary.

### 2.2. Propagation of Tissue-Resident Mø by Mixed Culturing with Interstitial Cells Obtained from the Testes

Mice testes were aseptically dissected and dipped into ice-cold HBSS immediately. Testicular tissue-resident Mø were allowed to proliferate according to a previously established method with modifications [8]. After removing the tunica albuginea, the testes were transferred to 15 mL conical tubes containing 10 mL cell dispersion enzyme solution: 20 mM HEPES-buffered HBSS (pH 7.4), 0.5 mg/mL collagenase type IA (C9891, Sigma-Aldrich), and 1 mM CaCl_2_. Testicular tissues were further digested at 37 °C for 20 min with gentle stirring at 120 rpm. After leaving the tubes to stand for a few minutes, during which almost all seminiferous tubules were deposited at the bottom, the supernatants including interstitial cells were transferred to 15 mL tubes and sedimented at 100× *g* for 5 min (Model 2410, Kubota, Tokyo, Japan). The testicular interstitial cells per mouse were plated in a 10 cm tissue culture dish (3020-100, AGC Techno Glass, Haibara, Japan) containing DMEM (D6046, Sigma-Aldrich), supplemented with 10% fetal bovine serum (FBS) (175012, Nichirei Biosciences, Tokyo, Japan), 100 U/mL penicillin, and 100 μg/mL streptomycin (pen/strep; P4333, Sigma-Aldrich) (DMEM-FBS), and further cultured in a CO_2_ incubator. The medium was refreshed every 3 days until multi-layered cells composed of Mø and other interstitial cells covered the dish surface. The over-confluent cells were then detached using 0.1% trypsin/2 mM ethylenediaminetetraacetic acid (EDTA) in HBSS and pipetted to dissociate the cells. Subsequently, the cells were used for the separation of testicular tissue-resident Mø. In some experiments, cells were subcultured or frozen at −80 °C at a dilution of 1:3 in a cell suspension with Bambanker (CS-02-001, Nippon Genetics, Tokyo, Japan) as a cryopreservative, and then maintained in the same medium until they regained over-confluence.

### 2.3. Separation of Testicular Tissue-Resident Mø Propagated by Mixed Culture from Interstitial Cells

Mixed-cultured testicular tissue-resident Mø were separated from other interstitial cells using a previously reported method [8]. The over-confluent cells collected from a 10 cm tissue culture dish were seeded in a 5.5 cm bacteriological Petri dish (1-8549-02, As One, Osaka, Japan) containing DMEM-FBS. When the Mø selectively adhered onto the surface of the dish after several to twenty-four hours of culture and the other interstitial cells formed nonadherent cell aggregates in the dish, the dish was washed with conditioned media to remove nonadherent cells. Subsequently, the adherent cells were detached using 5 mM EDTA in 10 mM HEPES-buffered HBSS (EDTA-HEPES-HBSS), followed by pipetting. The cell suspension was passed through a cell strainer (352235, BD Falcon, Franklin Lakes, NJ, USA) to remove cell aggregates and centrifuged at 220× *g* for 5 min, followed by suspension in HBSS (for reverse transcription-polymerase chain reaction (RT-PCR), quantitative PCR (qPCR), and enzyme-linked immunosorbent assay (ELISA)) or Ca/Mg-free phosphate-buffered saline (PBS; 1102P10, Cell Science & Technology Institute, Yamagata, Japan) containing 1% bovine serum albumin (BSA; A3059, Sigma-Aldrich) and 2 mM EDTA (BSA/EDTA-PBS; for phagocytosis analysis and flow cytometry). The cell count was measured and the cells were then used for further experiments.

Testicular interstitial cells mixed with testicular Mø were purified by treatment with clodronate-encapsulating liposomes to deplete phagocytic cells. Forty microliters of clodronate-encapsulating anionic liposomes (Clophosome-A, 7 mg of clodronate/mL, F70101C-A, FormuMax Scientific, Sunnyvale, CA, USA) were added to the mixed-cultured/over-confluent cells in six-well plates containing 2 mL DMEM-FBS per well. At 48 h following the addition, the medium was replaced with fresh medium containing clodronate liposomes. At 24 h following the second addition, the interstitial cells were washed thrice with HBSS and further used for RT-PCR expression analyses to examine the properties of conditioned testicular Mø.

### 2.4. Phagocytosis Analysis with Fluorescent Beads

We examined the phagocytic properties of testicular tissue-resident Mø in mixed culturing according to the method reported by Ogawa et al. [8] with modifications. After separation on the bacteriological Petri dish, 2.5 × 10^5^ cells/0.5 mL DMEM-FBS were placed in a 5 mL tube that was siliconized to prevent adhesion to the tube wall (Siliconise L-25, 0411002, Fuji-Rika Industries, Osaka, Japan). After 1 µL of fluorescent yellow-green-conjugated latex beads (mean diameter, 1 µm; L4655, Sigma-Aldrich) were added, the cells were incubated at 37 °C for 1 h with gentle shaking at 18 rpm on a see-saw rocking shaker (Wave SI slim; Taitec, Koshigaya, Japan). The cells were then washed thrice with HBSS and plated on a 3.5 cm glass-bottom dish (3910-035, AGC Techno Glass) with 1.5 mL DMEM-FBS for approximately 1 h until almost all the cells had adhered to the surface. After fixation with 10% formalin (Kanto Chemical, Tokyo, Japan) in PBS for more than 10 min, green fluorescence, phase-contrast, and differential interference images of the same fields were captured using a 10× and 20× objective lens (IX71; Olympus, Tokyo, Japan). Cells that engulfed more than two latex beads were denoted as Mø. We counted >700 cells in each sample, and the percentage of Mø in each mouse was calculated from independent experiments (three mice and three experiments for the testicular cells). Data are presented as the mean ± SD.

### 2.5. Flow Cytometry

Flow cytometry was performed to examine the expression of nine Mø/monocyte markers in testicular tissue-resident Mø in mixed culture and separated using bacteriological Petri dishes according to the method described by Ogawa et al. [8] with modifications. The monoclonal antibodies used in the flow cytometric analyses are listed in Supplementary Table S1. Cells at a density of approximately 1 × 10^6^/0.5 mL of BSA/EDTA-PBS were fixed in 10% formalin in BSA/EDTA-PBS for approximately 20 min at 24 °C. After washing with BSA/EDTA-PBS, the cells were permeabilized using 0.2% saponin (30502-42, Nacalai Tesque, Kyoto, Japan) in 1 mL BSA/EDTA-PBS for 5 min at 24 °C. To avoid non-specific Fc-gamma receptor-mediated binding of fluorochrome-conjugated antibodies, approximately 2.5 × 10^5^ cells/50 µL of the cell suspensions were pretreated with 0.5 µg of anti-mouse CD16/32 antibody for 10 min at 24 °C. To the 50-µL cell suspension, 0.5 µg FITC-conjugated anti-CD11b, 0.15 µg FITC-conjugated anti-CD68, 0.5 µg FITC-conjugated anti-CD115, 0.1 µg APC-conjugated anti-CD116, 0.15 µg APC-conjugated anti-CD169, 0.25 µg APC-conjugated anti-CD206, 0.5 µg APC-conjugated anti-F4/80, 0.15 µg APC-conjugated anti-Mertk, and 0.25 µg APC-conjugated anti-MHC II were added as per the manufacturer’s instructions, and then incubated for 20 min at 4 °C. After washing, 20,000 cells were analyzed using flow cytometry (S3 Cell Sorter; Bio-Rad Laboratories, Hercules, CA, USA) for their expression characteristics. As controls, we used cell suspensions that were pretreated with the anti-mouse CD16/32 antibody and treated with the same quantity of fluorochrome-labeled isotype control antibody as the test antibody. The expression of marker molecules in Mø was determined from more than three independent experiments using testicular tissues derived from more than three mice.

### 2.6. Immunofluorescence Staining

The testes were fixed using 4% paraformaldehyde in PBS for 3–4 h at 4 °C. After washing with PBS, the tissues were immersed in 30% sucrose in PBS overnight and mounted in optimal cutting temperature compound (4583, Sakura Finetechnical, Tokyo, Japan); 6-µm-thick cryostat sections were used for immunofluorescence staining. 

CD206 (rat monoclonal antibody, MR6F3, 17-2061-80, Thermo Fisher Scientific, Waltham, MA, USA) and F4/80 (rat monoclonal antibody, BM8; BMA Biomedicals, Augst, Switzerland) antibodies were used to examine the localization of testicular tissue-resident Mø. An HSD3B (rabbit polyclonal antibody; KO607, TransGenic, Kobe, Japan) antibody was used to label steroidogenic cells, such as Leydig cells. GJA1 antibody (rabbit polyclonal antibody; C6219, Sigma-Aldrich) was used to identify gap junctions. Double-immunofluorescence staining was performed as previously described [20]. Cryostat sections were incubated with a mixture of 1 µg/mL rat anti-CD206 and 1.5 µg/mL rabbit anti-GJA1 antibody or 1.5 µg/mL rat anti-F4/80 and 1 µg/mL rabbit anti-HSD3B in PBS containing 1% BSA (BSA-PBS; A3059, Sigma-Aldrich) for 90 min at 32 °C. After washing with PBS, the sections were incubated with a mixture of 5 µg/mL Alexa Fluor 488-conjugated donkey anti-rabbit IgG (A21206, Thermo Fisher Scientific) and 5 µg/mL Alexa Fluor 594-conjugated donkey anti-rat IgG (A21209, Thermo Fisher Scientific) in BSA-PBS for 30 min at 32 °C. After washing with PBS and mounting with PermaFluor (TA-030-FM, Thermo Fisher Scientific), the sections were photographed under a fluorescence microscope (IX71; Olympus). Some sections were also stained with 4′,6-diamidino-2-phenylindole dihydrochloride (DAPI, 2 µg/mL; 342-07431, FUJIFILM Wako Chemical, Tokyo, Japan), which was included in the mixture of secondary antibodies.

### 2.7. Total RNA Extraction and RT-PCR Analyses

Total RNA was isolated from the testes, testicular tissue-resident Mø, and interstitial cells in mixed culture using the TRI Reagent (TR118, Molecular Research Centre, Cincinnati, OH, USA); RT-PCR analysis was performed as previously described [21,22]. Total RNA (1 μg) was transcribed into first-strand cDNA using Moloney Murine Leukemia Virus (M-MLV) RT, RNase H^−^ (316-08151, Nippon Gene, Toyama, Japan), and an oligo (dT)_18_ primer, as per the manufacturer’s instructions. To examine the transcript expression, 0.5 µL of a 25-µL reaction mixture was amplified with Taq DNA polymerase (TaKaRa Ex Taq HS, RR006A; TaKaRa Bio, Otsu, Japan) using the reverse-transcribed cDNA as the template. The primer pairs and thermal cycling conditions used for PCR amplification in this study are listed in Supplementary Tables S2 and S3. The RT reaction was not performed for negative controls. The PCR products were separated on 1.5% agarose gels and visualized using GelRed (41002, Biotium, Inc., Fremont, CA, USA) or ethidium bromide staining. The expression of transcripts was determined from more than three independent experiments in the testes and testicular primary cells derived from more than three mice.

### 2.8. P4 Measurement

Propagated testicular Mø were seeded at 4 × 10^5^ cells/well (six wells; 2 mL DME-FBS/well) and incubated at 37 °C in a CO_2_ incubator. The culture medium at 24 h and/or 72 h after seeding was collected, and P4 concentrations in the medium were measured using an ELISA kit (P4 ELISA kit; ADI-900-011 Enzo Life Sciences, Farmingdale, NY, USA) according to the manufacturer’s protocol. To demonstrate P4 production specific to testicular Mø, liver, lung, and spleen tissue-resident Mø propagated similarly in mixed culture with interstitial cells of the respective organs from ICR male mice, were seeded under the same conditions, and their culture media collected at 24 h after seeding were assayed. In addition to untreated controls, the testicular Mø cultured with DME-FBS containing the following reagents were assayed: (1) 50 µM dibutyryl cAMP (D0627, Sigma-Aldrich); (2) 1 µM isoproterenol (Iso, I6504, Sigma-Aldrich); (3) 1 µM Iso plus 100 nM β2-adrenergic antagonist ICI-118551 (ICI; S8114, Selleck Chemicals, Houston, TX, USA); and (4) 20 ng/mL lipopolysaccharide (LPS; L4391, Sigma-Aldrich) plus 50 ng/mL interferon-γ (IFN-γ; AF-315-05-100UG, PeproTech, Rocky Hill, NJ, USA) as Mø polarization inducers of the classic M1 phenotype. P4 concentrations were determined from more than three independent experiments in Mø derived from more than three mice. P4 concentrations are presented as mean ± SD, and concentration levels of P4 relative to untreated controls were calculated to clearly demonstrate differences. After the collection of the culture medium, the testicular Mø were used for qPCR analyses. 

Statistical analyses were performed using Microsoft Excel and with statistical software available online (http://statpages.info/anova1sm.html, accessed on 27 September 2021). Differences between and among groups were evaluated by Student’s *t*-test and/or one-way ANOVA, followed by Tukey’s honest significant difference post hoc analysis. Statistical significance was set at a *p*-value of < 0.05. All values represent mean ± SD and are expressed relative to the average control value, which is set as 1.00.

### 2.9. qPCR Analysis

The cDNA synthesized for RT-PCR was used as the template for qPCR analyses. qPCR was conducted using GeneAce SYBR qPCR Mix αLow ROX (316-07693, Nippon Genetics, Tokyo) and a qPCR machine (Applied Biosystems 7500, Thermo Fisher Scientific), according to the manufacturer’s instructions and protocol. The primers and reaction conditions for qPCR are listed in Supplementary Table S4. Reactions were performed as follows: Preheating at 95 °C for 10 min, followed by 45 cycles of amplification at 95 °C for 30 s and 60 °C for 1 min. The mRNA expression data were calculated using the comparative CT method (2^−∆∆CT^ method). *Actb* was used as an endogenous control to normalize expression levels. Relative expression levels were determined from more than four independent experiments in testicular Mø derived from more than four mice and are presented as the mean ± SD.

## 3. Results

### 3.1. Propagation of Testicular Tissue-Resident Mø in Mixed Culture with Interstitial Cells Rich in Leydig Cells and Segregation of Mø by Adhesion to the Bacteriological Petri Dish

**Figure 1:**
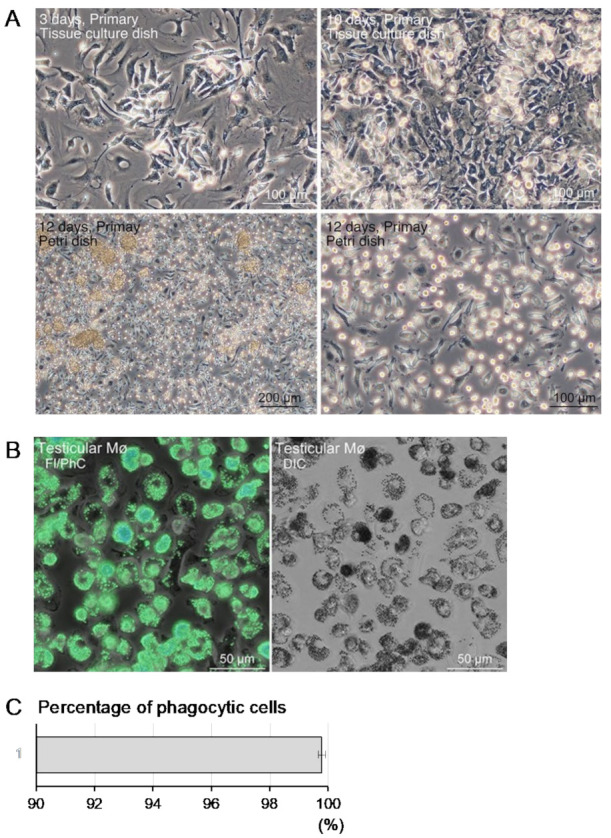
Propagation and separation of testicular macrophages (Mø) in co-culture with testicular interstitial cells and their phagocytic property. Testicular interstitial cells from two testes of one mouse were collected by dispersing the cells with collagenase, seeding in a 10 cm tissue culture dish, and culturing with standard culture medium. Testicular interstitial cells propagated and reached over-confluence within 10 days. Cells harvested from an over-confluent dish were seeded in a bacteriological Petri dish to separate highly adhesive Mø from other interstitial cells. (**A**) Phase-contrast images of primary testicular interstitial cells cultured in a tissue culture dish for the indicated days (upper panels). Testicular tissue-resident Mø selectively adhere to the Petri dish surface (lower panels), and nonadherent cells formed cell aggregates (lower left panel), which were easily removed by tapping and washing with conditioned medium (lower right panel). (**B**) Phagocytotic properties assessed by incubation with fluorescent beads in testicular Mø segregated by adhesion to bacteriological Petri dishes. A green fluorescence image (Fl) merged with the phase-contrast image (PhC) (left panel) and differential interference contrast (DIC) images of the same fields (right panel). (**C**) Bar graph presenting the percentage of testicular tissue-resident Mø in the cells adherent to the bacteriological Petri dishes, presented as mean ± SD. More than 700 cells per sample were counted, and the percentage of phagocytic cells from three mice was determined from three independent experiments.

Testicular tissue-resident Mø were propagated by mixed culture with interstitial cells from mouse testis. Testicular Mø revealed high propagation in standard culture medium without additional growth factors for Mø such as CSF1. Primary testicular interstitial cells, including Mø, reached over-confluence within 10 days (Figure 1A) by changing the culture medium every 3 days. Over-confluent cells were allowed to form a multilayered structure on a standard tissue culture dish. Mø were identified as small flat/elongated cells with a few cytoplasmic protrusions, small round or fusiform cells, and mixed-cultured interstitial cells.

The propagated Mø were separated from the other interstitial cells in the mixed culture because only the Mø adhered to the bacteriological Petri dish. Small round/fusiform cells with a few cytoplasmic protrusions, identified as Mø, adhered to the dish surface within several hours of seeding the mixed culture with over-confluent cells, as evidenced by cell aggregates floating in the medium (Figure 1A). These cell aggregates were easily eliminated by washing with conditioned medium. Adherent cells (>1.6 × 10^6^) were collected from every 6 cm bacteriological Petri dish and their Mø features were analyzed. The over-confluent mixed culture cells were sub-cultured or frozen at a cell dilution ratio of 1:3. The sub-cultured cells, and thawed and cultured frozen cells, had the same culture conditions. These cells proliferated slowly and generally reached over-confluence after >10 days. Thus, primary cells were used in most experiments.

Phagocytosis of fluorescent beads was assessed to precisely determine the percentage of Mø in the segregated cells that had adhered to the bacteriological Petri dishes. During incubation, almost all cells that had adhered to the Petri dish phagocytosed the fluorescent beads (Figure 1B). Most cells contained multiple beads in their cytoplasm. This observation shows that Mø in the mixed culture possessed a high phagocytic property. Bead-positive and bead-negative cells were counted to estimate the percentage of Mø in the segregated cells. We defined the cells that phagocytosed more than two beads as bead-positive cells and counted >700 such cells in every sample. Overall, these cells comprised 99.8% ± 0.1% of the entire population (Figure 1C). Thus, segregating Mø according to their degree of adhesion to the bacteriological Petri dish is a reliable and simple method for isolating testicular Mø from other interstitial cells in the mixed culture. 

### 3.2. Mø Marker Expression Profiles by Flow Cytometry in Propagated Testicular Mø with the Mixed Culture

**Figure 2:**
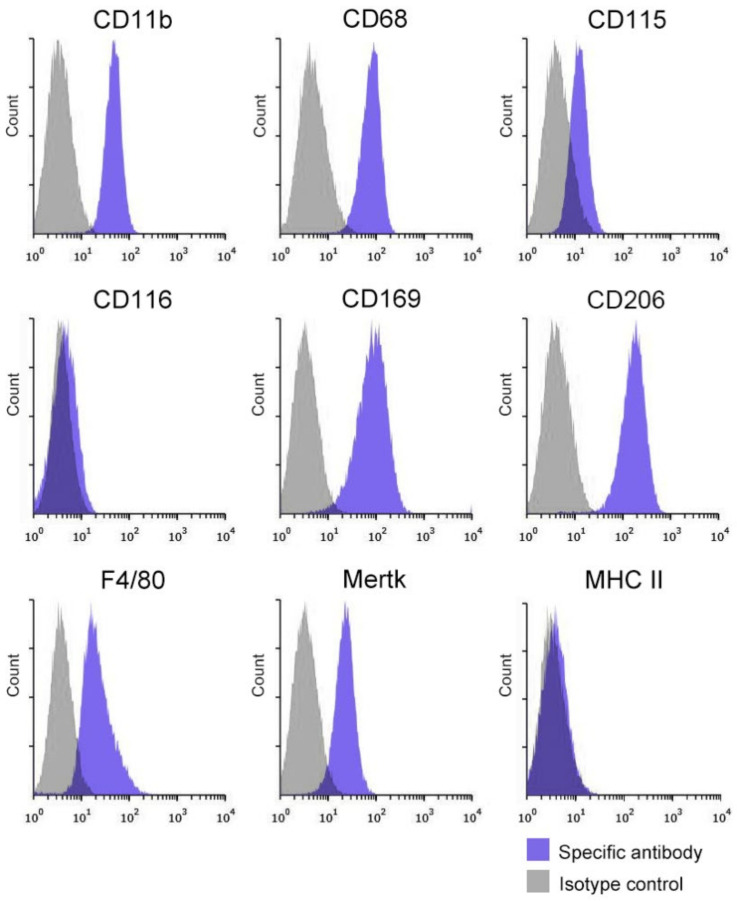
Macrophage (Mø) marker expressions in propagated Mø identical to properties of testicular interstitial Mø. Representative histograms from flow cytometric analyses revealing the expression of nine Mø/monocyte markers in propagated testicular Mø by mixed culture (blue histogram, specific antibody; grey histogram, isotype control). Cell suspensions were pretreated with an anti-mouse CD16/32 antibody and further treated with a fluorochrome-labeled test antibody, or the same amount of fluorochrome-labeled isotype control antibody. Propagated testicular Mø present characteristic expression patterns of highly CD206-positive and MHC II-negative cells, which are identical to those of testicular interstitial Mø.

The expression profiles of nine Mø markers in propagated testicular Mø segregated from interstitial cells were examined by flow cytometry. The propagated Mø were composed of a single fraction based on the histograms of the marker expression distributions. Testicular Mø revealed high expressions of CD11b, CD68, CD169, and CD206; substantial/clear expressions of CD115, F4/80, and Mertk; and almost no expressions of CD116 and MHC II (Figure 2). The propagated testicular Mø were highly CD11b-positive, CD115-positive, highly CD206-positive, CD116-negative, and MHC II-negative, suggesting interstitial Mø [11,18,23].

### 3.3. Expression Profiles of Transcription Factors Shaping Mø and Molecules Involved in Spermatogenesis and Mø Properties in Propagated Testicular Mø

**Figure 3:**
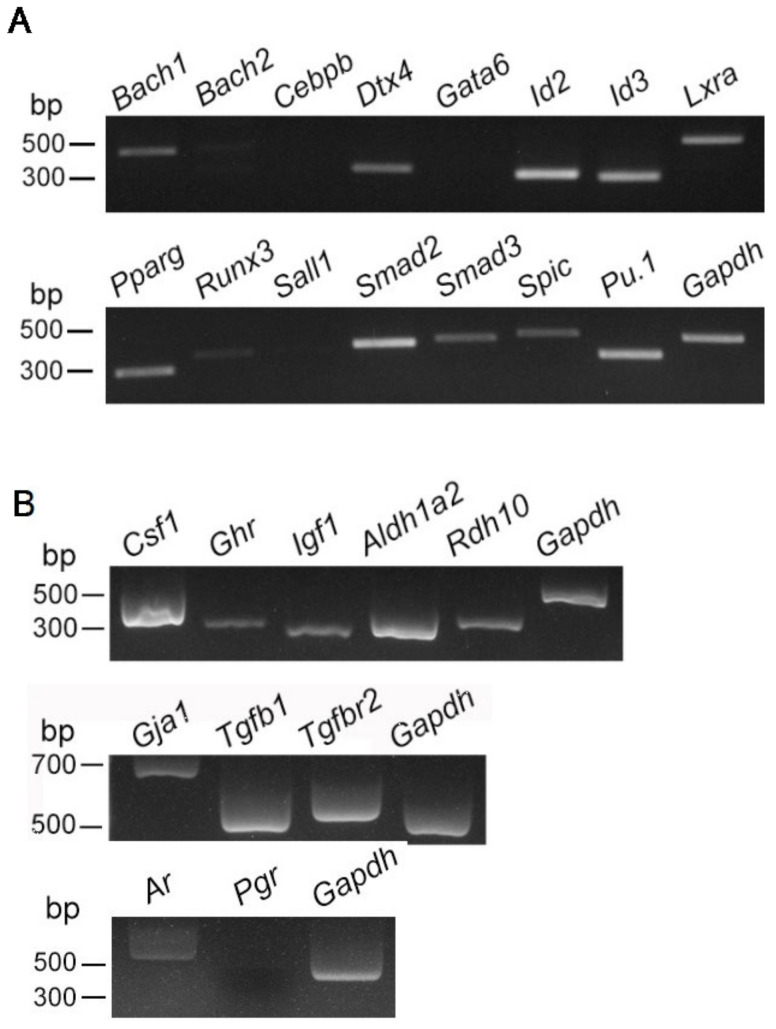
Expression profiles of transcription factors and molecules characterizing testicular tissue-resident macrophages (Mø) propagated by mixed culture. mRNA expression in primary Mø propagated by mixed culture with interstitial cells was analyzed using a reverse transcription-polymerase chain reaction (RT-PCR). (**A**) Expression profiles of 14 transcription factors that are tissue-/organ-specific for resident Mø and the lineage-determining transcription factor of Mø, *Pu.1*, in propagated testicular Mø. (**B**) Expression profiles of molecules characterizing testicular tissue-resident Mø in propagated Mø by mixed culture.

It has been reported that some transcription factors are resident tissue-/organ-specific in several representative tissue-resident Mø (BACH2, CEBPβ, PPARγ: alveolar Mø; DTX4, RUNX3: intestinal Mø; ID3, LXRα, SPIC: Kupffer cells; ID2, RUNX3: Langerhans cells; SALL1, SMAD2, SMAD3: microglia; CEBPβ, GATA6: peritoneal Mø; and BACH1, SPIC: red pulp Mø) [24,25]. Therefore, the mRNA expression analysis of these 14 transcription factors and a lineage-determining transcription factor of Mø PU.1 [25] was conducted in propagated testicular Mø. We found that the testicular Mø expressed *Bach1*, *Dtx4*, *Id2*, *Id3*, *Lxra*, *Pparg*, *Runx3*, *Smad2*, *Smad3*, *Spic*, and *Pu.1*, but *Runx3* expression was very low, as assessed by RT-PCR (32 cycles, Figure 3A).

Previous studies have also revealed that (i) testicular interstitial cells and/or tissue-resident Mø produce CSF1, IGF1, and retinoic acid (RA) [10,12,26] along with AR and PGR [27,28], which are implicated in spermatogenesis, and (ii) certain tissue-resident Mø express GJA1 [29,30] along with TGFB1 and TGFBR2 [21,31]. Thus, we examined the expression of these molecules and RA synthetic enzymes, i.e., ALDHLA2 and RDH10, in Mø by RT-PCR. We observed that the propagated Mø clearly expressed *Csf1*, *Ghr*, *Igf1*, *Aldhla2*, and *Rdh10* (Figure 3B). Moreover, they expressed *Gja1*, *Tgfb1*, *Tgfbr2*, and *Ar*. These findings indicate that propagated Mø likely maintain testicular Mø-specific properties to modulate spermatogenesis and communicate with Leydig cells.

### 3.4. Properties of Testicular Interstitial Cells Propagated by Mixed Culture with Testicular Mø

**Figure 4:**
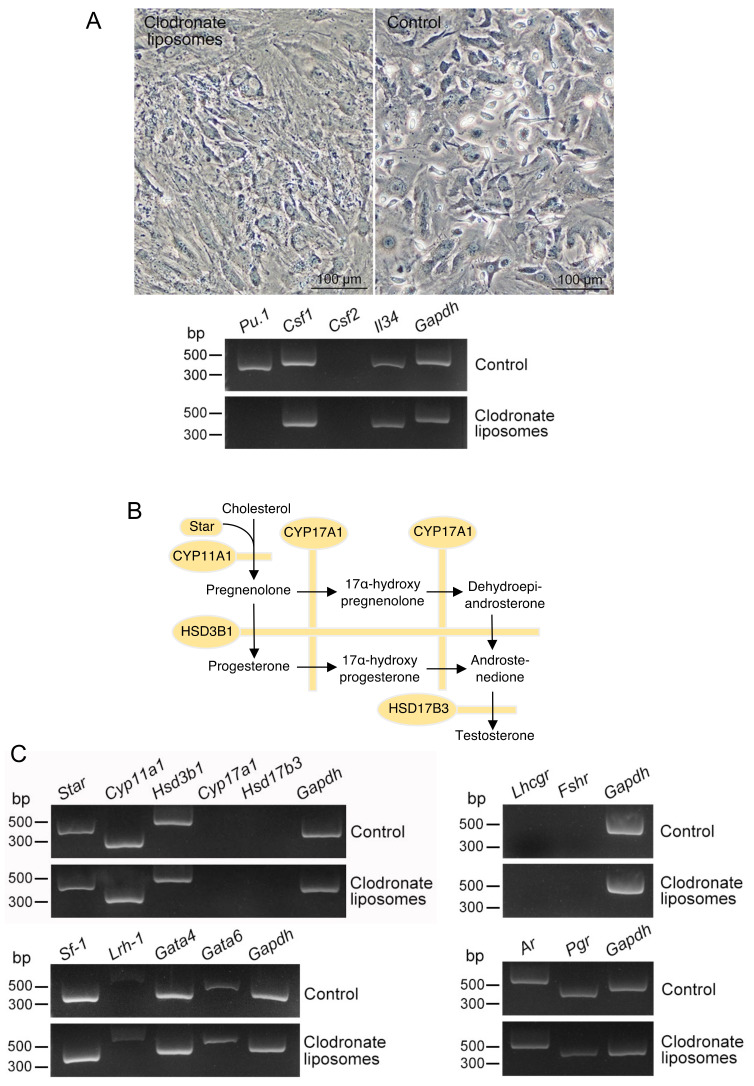
Properties of testicular interstitial cells propagated in mixed culture and depletion of phagocytic cells. Testicular interstitial cells were propagated by mixed culture, treated with clodronate liposomes for 3 days to deplete testicular macrophages (Mø), and used as samples to examine the expression of molecules related to Mø propagation and sex steroid production by reverse transcription-polymerase chain reaction (RT-PCR). (**A**) Phase-contrast images (upper panel) and expression profiles of growth factors for Mø (lower panel) in testicular interstitial cells propagated by mixed culture and treated with or without clodronate liposomes. Small round/oval/fusiform cells, identical to the morphological features of Mø, disappeared in the interstitial cells treated with clodronate liposomes. These cells do not express *Pu.1* as a universal marker of Mø, albeit they clearly express *Csf1* and *Il34*. (**B**) Schematic drawing demonstrating pathways and molecules involved in androgen synthesis from cholesterol. (**C**) Expression profiles of molecules involved in androgen production by RT-PCR in the interstitial cells propagated by mixed culture and treated with or without clodronate liposomes.

Testicular Mø were propagated along with interstitial cells in the mixed culture. We hypothesized that propagated interstitial cells serve as the niche cells for conditioning testicular Mø; thus, we examined the niche properties of the testicular interstitial cells. After treatment with clodronate-encapsulating liposomes for Mø depletion, almost all small round/fusiform cells, i.e., Mø, disappeared in the mixed culture, as assessed by microscopy (Figure 4A). The mixed-cultured interstitial cells treated with the reagent did not express *Pu.1*, whereas the untreated cells clearly expressed *Pu.1*, the critical master regulator in Mø differentiation/development [25]. Moreover, mixed-cultured cells treated with the reagent clearly expressed *Csf1* and *Il34*, but they did not express *Csf2* (Figure 4A), which induces the proliferation of tissue-resident Mø [5]. 

Testicular interstitial Mø derived from embryonic precursors are located among Leydig cells [11,18]; thus, we hypothesized that Leydig cells form the niche of interstitial Mø not only in vivo but also in mixed cultures. We determined whether the propagated mixed-cultured cells were composed of Leydig cells by examining the expression of molecules essential for de novo testosterone synthesis (Figure 4B), transcription factors regulating the expression of these molecules [32,33,34], and steroid hormone receptors. Using RT-PCR, we observed that mixed-cultured interstitial cells treated with clodronate liposomes expressed *Star*, *Cyp11a1*, and *Hsd3b1*, but not *Cyp17a1* or *Hsd17b3*. Moreover, they expressed *Sf-1*, *Lrh-1*, *Gata4*, *Gata6*, *Ar*, and *Pgr* (Figure 4C). These results suggest that the mixed-cultured interstitial cells were mainly composed of steroidogenic cells which are partially similar to Leydig cells, which likely lost testosterone synthetic properties, albeit maintained the de novo synthesis of P4. This is consistent with previous studies that demonstrated that Leydig cells in primary cultures immediately lose testosterone production properties [35,36]. 

### 3.5. Localization of Testicular Mø Exhibiting Properties of Steroid Production

**Figure 5:**
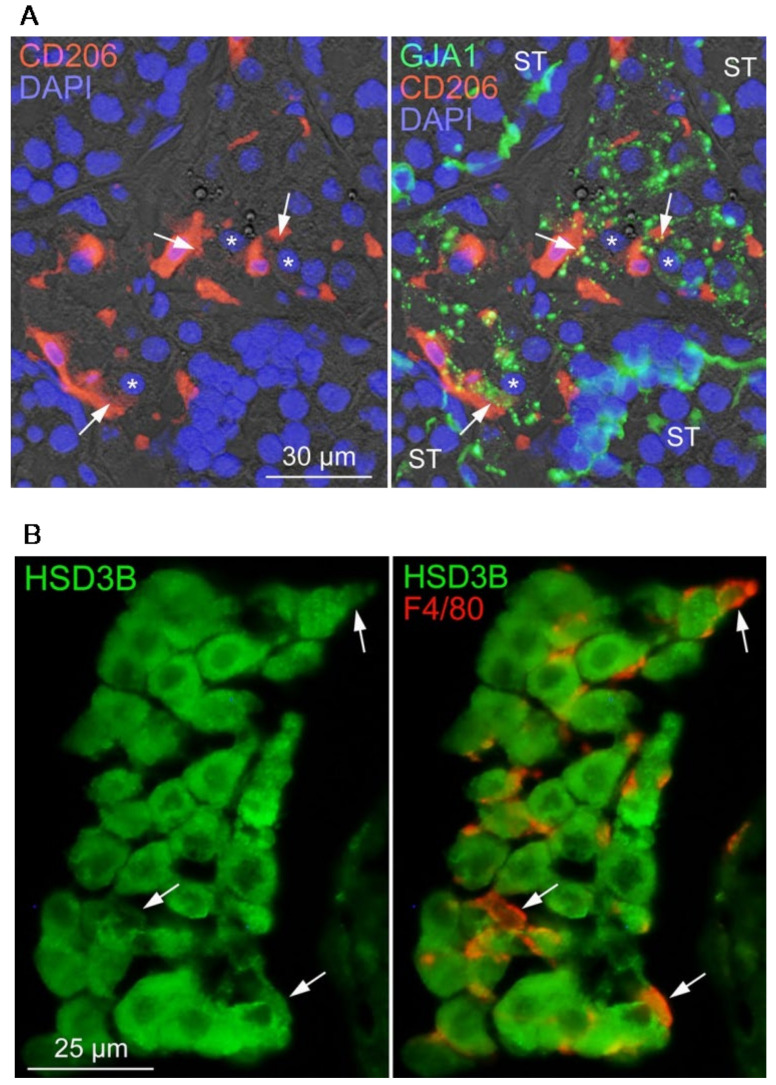
Gap junction localization between interstitial macrophages (Mø) and Leydig cells and HSD3B expression in interstitial Mø. (**A**) Immunofluorescence micrographs demonstrating the distribution of gap junctions in the adult mouse testis. Fluorescence images are merged with differential interference contrast images. Cryostat sections were stained with the indicated antibodies and 4′,6-diamidino-2-phenylindole (DAPI) (blue). Punctate GJA1 immunoreactivity (green) is localized in CD206-positive (red) interstitial Mø and interstitial cells (arrows) with a large round nucleus (*), an identical profile of Leydig cells. ST, seminiferous tubule. (**B**) Immunofluorescence micrographs demonstrating HSD3B immunoreactivity (green) in interstitial cells. HSD3B immunoreactivity is localized not only in Leydig cells but also weakly in F4/80-positive interstitial Mø (arrows).

We observed that propagated testicular Mø express CD206, F4/80, and *Gja1* (Figure 2 and Figure 3B). Previous studies have revealed that Leydig cells also express GJA1 [37] and testicular tissue-resident Mø can produce 25-hydroxycholesterol, a substrate of testosterone production [38]. Thus, we examined immunohistochemically whether GJA1 and HSD3B (steroid synthetase) were expressed in testicular Mø in vivo. We observed that punctate GJA1 immunoreactivity was localized between CD206-positive Mø and CD206-negative cells with a round/large nucleus, indicating a feature of Leydig cells, in the testicular interstitium (Figure 5A). We also observed that HSD3B immunoreactivity was localized strongly in F4/80-negative cells, i.e., Leydig cells, and weakly in F4/80-positive interstitial Mø (Figure 5B). These findings indicate that gap junctions may be formed between testicular interstitial Mø and Leydig cells, and testicular interstitial Mø may possess the property of de novo steroidogenesis.

### 3.6. P4 production in Testicular Mø Propagated in Mixed Culture

**Figure 6:**
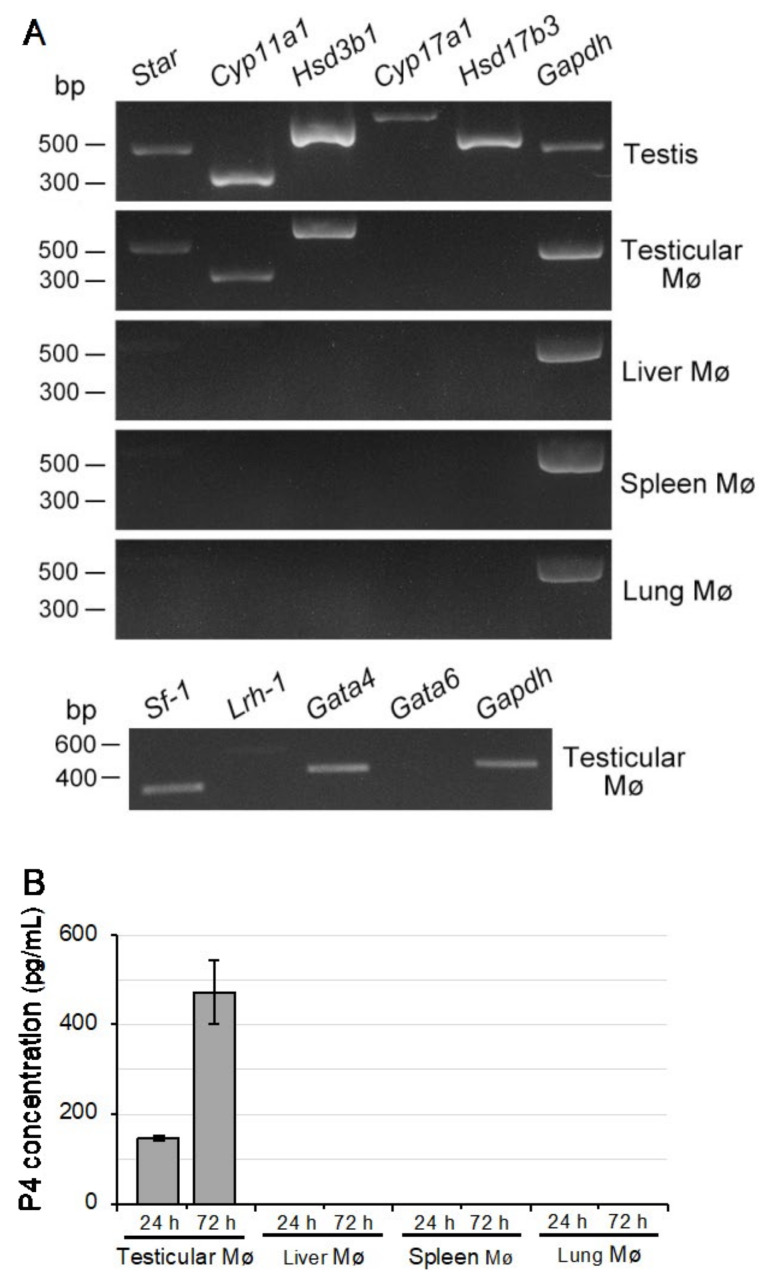
Progesterone (P4) is produced de novo specifically in testicular macrophages (Mø), albeit not in the liver, spleen, and lung Mø that are propagated by mixed culture. (**A**) Reverse transcription-polymerase chain reaction (RT-PCR) amplification of molecules related to androgen production. *Star*, *Cyp11a1*, and *Hsd3b1* are clearly expressed in testicular Mø, albeit not in the liver, spleen, and lung tissue-resident Mø that are propagated by mixed culture with the respective organ-specific cells. Testicular Mø also express *Sf-1* and *Gata4*. (**B**) P4 concentrations in the cultured testis, liver, spleen, or lung Mø. Mø propagated by mixed culture were seeded at a density of 4 × 10^5^ cells/2 mL in six-well plates, and the medium cultured for 24 h and 72 h was collected and assayed for P4 concentrations by ELISA. P4 concentrations were determined from three independent experiments in propagated testicular Mø derived from three mice. P4 was detected only in the testicular Mø samples. Data are presented as the mean ± SD.

As we immunohistochemically identified HSD3B expression in testicular Mø in the interstitium, we further examined the mRNA expression of the molecules essential for de novo testosterone synthesis in the propagated testicular Mø. RT-PCR analysis revealed that the testicular Mø expressed *Star*, *Cyp11a1*, and *Hsd3b1*, whereas the liver, spleen, and lung Mø, which propagated similarly in mixed culture with organ-specific interstitial cells, did not express all these molecules (Figure 6A). These findings suggest that testicular Mø synthesize sex steroids de novo up to P4 (Figure 4B). The nuclear receptors SF-1, LRH-1, GATA4, and GATA6 are transcription factors that are highly expressed in steroidogenic tissues and they upregulate molecules involved in steroid hormone synthesis [32,33,34]. Thus, we examined the expression of these transcription factors by RT-PCR and observed that *Sf-1* and *Gata4* were clearly expressed in the propagated testicular Mø (Figure 6A). These findings suggest that propagated testicular Mø possess the properties of steroidogenic cells. Thus, we further examined de novo P4 production in propagated testicular Mø using ELISA of conditioned culture medium. P4 concentrations in conditioned medium-cultured testicular Mø at 4 × 10^5^ cells/2 mL for 24 h and 72 h were 146.6 ± 5.2 and 472.0 ± 70.8 pg/mL, respectively; however, P4 was not detected in cultured liver, spleen, and lung Mø that were propagated similarly in mixed culture (Figure 6B). P4 concentrations in the fresh culture medium as a negative control were below the detection limit (8.6 pg/mL; data not presented). These findings indicate that the P4 concentration increase is correlated with culture periods and testicular Mø possess organ-specific properties for P4 de novo synthesis.

### 3.7. P4 Production Upregulation in Testicular Mø by cAMP and Iso

**Figure 7:**
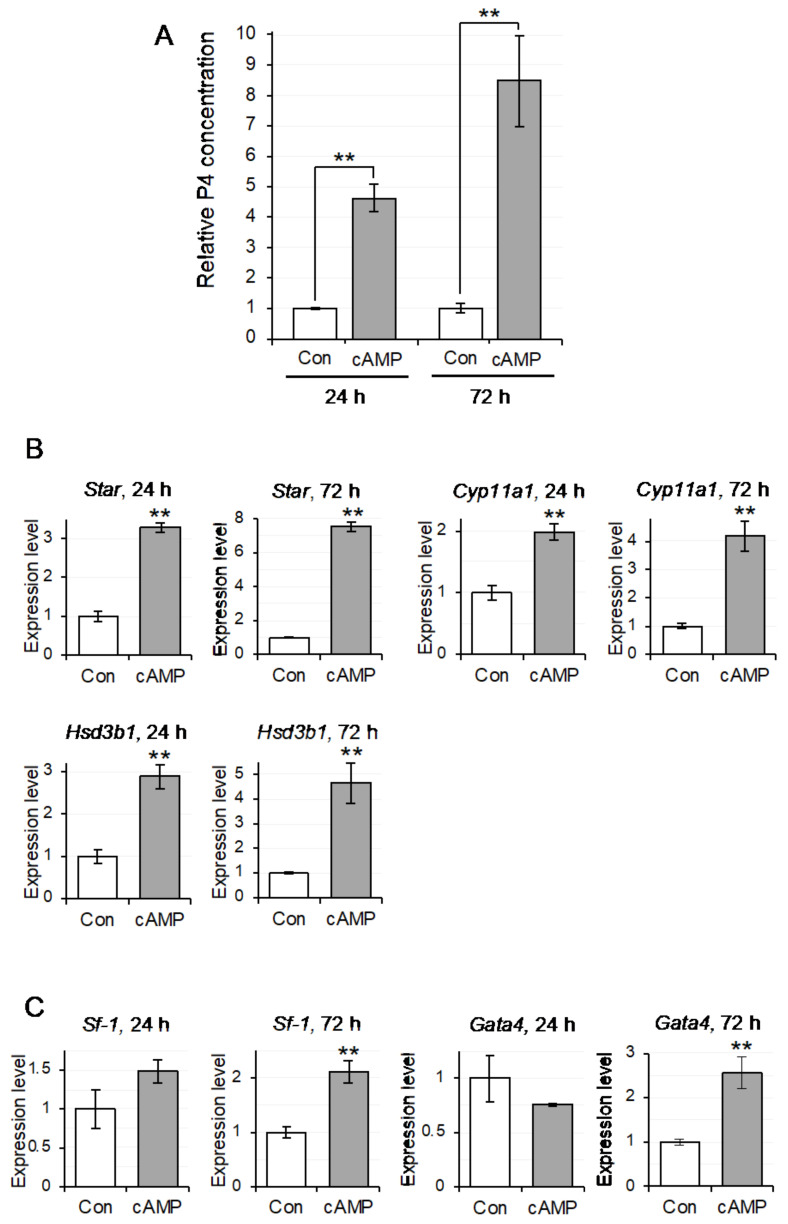
Cyclic adenosine monophosphate (cAMP) induces upregulation of progesterone (P4) production and molecules related to P4 production in testicular macrophages (Mø). Testicular macrophages (Mø) were seeded at a density of 4 × 10^5^ cells/2 mL in six-well plates and cultured in DMEM-FBS with (cAMP) or without (Con) 50 µM dibutyryl cAMP. P4 concentration in the medium and molecules involved in P4 production were measured using enzyme-linked immunosorbent assay (ELISA) and quantitative polymerase chain reaction (qPCR), respectively, in the testicular Mø at 24 h and 72 h after addition. Data were determined from three independent experiments in propagated testicular Mø derived from three mice and are presented as mean ± SD (** *p <* 0.01). (**A**) Relative P4 concentration in the culture medium of testicular Mø treated with or without dibutyryl cAMP. (**B**,**C**) The expression levels of *Star*, *Cyp11a1*, and *Hsd3b1* as well as *Sf-1* and *Gata4* in the testicular Mø treated with (cAMP) or without (Con) dibutyryl cAMP were determined by qPCR with the 2^−∆∆CT^ method and are normalized to those of *Actb*. The mRNA expression levels relative to the untreated controls were upregulated and significantly different between the two groups in most cases.

Our immunohistochemical findings suggested that gap junctions were formed between the testicular Mø and Leydig cells. Testosterone production in Leydig cells is upregulated by an increase in intracellular cAMP concentration induced by luteinizing hormone (LH) stimulation [39]. Therefore, we hypothesized that cAMP in Leydig cells enters adjacent Mø through the gap junctions, and we examined the effects of cAMP on P4 production in testicular Mø using membrane-permeable dibutyryl cAMP. cAMP markedly increased P4 concentrations in the culture media. At 24 h and 72 h following the addition of dibutyryl cAMP, P4 concentrations were 676.6 ± 67.1 and 4003.3 ± 706.0 pg/mL, respectively; relative P4 concentrations to those in the untreated controls were 4.61 ± 0.46 and 8.48 ± 1.50 times higher, respectively, which was significantly different (*p* < 0.001 and *p* < 0.001, respectively) from the untreated controls (Figure 7A). The P4 concentration in the untreated controls at 72 h was 3.22 times higher than that at 24 h. These results indicate that cAMP upregulates P4 production in testicular Mø, and this effect is sustained for at least 3 days. To support these cAMP effects, we examined the mRNA expression of molecules involved in steroidogenesis by qPCR. cAMP upregulated the expression of *Star*, *Cyp11a1*, and *Hsd3b1* at 24 h and 72 h following dibutyryl cAMP addition, respectively, and that of *Sf-1* and *Gata4* at 72 h (Figure 7B,C). Expression levels of *Star*, *Cyp11a1*, *Hsd3b1*, *Sf-1*, and *Gata4* relative to the untreated controls at 24 h were 3.29 ± 0.14, 1.98 ± 0.15, 2.87 ± 0.33, 1.49 ± 0.16, and 0.76 ± 0.01, respectively, and were significantly different, except for those of *Sf-1* and *Gata4* (*p* < 0.001, *p* = 0.001, *p* =0.001, *p* = 0.082, and *p* < 0.181, respectively); expression levels of *Star*, *Cyp11a1*, *Hsd3b1*, *Sf-1*, and *Gata4* relative to the untreated controls at 72 h were 7.54 ± 0.36, 4.17 ± 0.46, 4.65 ± 0.99, 2.11 ± 0.24, and 2.56 ± 0.45, respectively and were significantly different (*p* < 0.001, *p* = 0.001, *p* = 0.003, *p* = 0.002, and *p* < 0.004, respectively). These findings indicate that upregulation of P4 production by cAMP in the testicular Mø appears to be dependent on the upregulation of these mRNA. 

**Figure 8:**
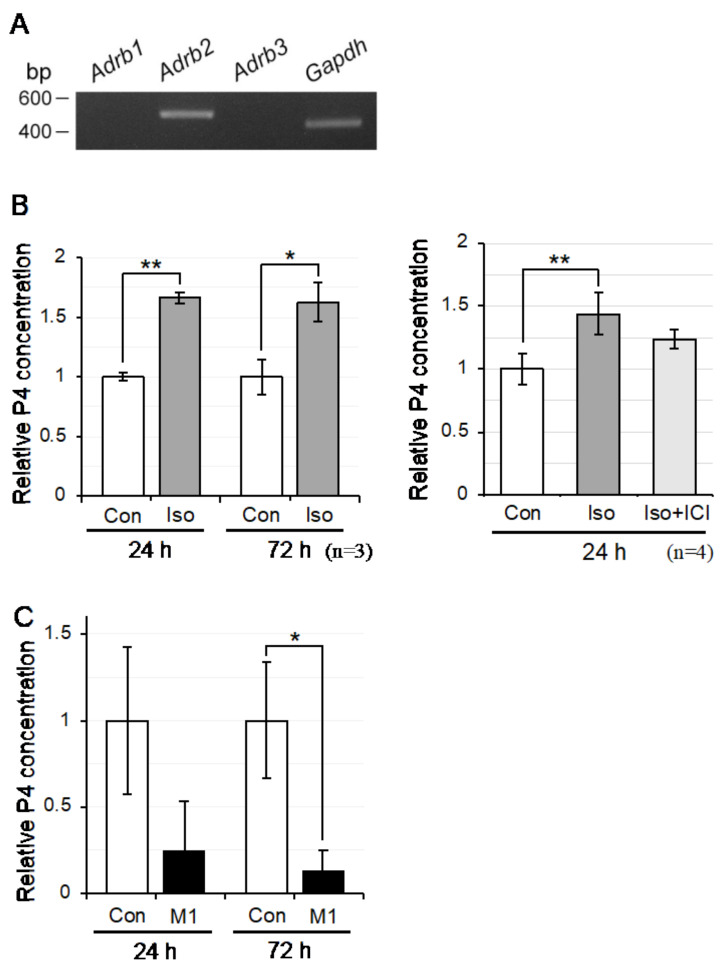
Progesterone (P4) production is upregulated by isoproterenol and inhibited by M1 polarization inducers in testicular macrophages (Mø). Testicular Mø were seeded at a density of 4 × 10^5^ cells/2 mL in six-well plates and cultured in DMEM-FBS containing 1 µM isoproterenol (Iso), 1 µM Iso plus 100 nM β2-adrenergic antagonist ICI-118551 (Iso + ICI), and 20 ng/mL lipopolysaccharide plus 50 ng/mL interferon-γ (M1). At 24 h and/or 72 h after the addition, the medium was collected, and P4 concentration was measured using an ELISA kit. P4 concentrations relative to the medium without reagents were calculated. Data were determined from more than three independent experiments in propagated testicular Mø derived from more than three mice and presented as mean ± SD (* *p* < 0.05; ** *p* < 0.01). (**A**) Expression of β-adrenergic receptors in testicular Mø were examined by reverse transcription-polymerase chain reaction (RT-PCR). (**B**) P4 production levels were enhanced by the addition of Iso. The relative P4 concentration levels in the Iso + ICI group tended to attenuate the enhancing effect of the Iso group (*p* = 0.076). (**C**) P4 concentration levels in the M1 group relative to the untreated controls (Con) were significantly different between the two groups at 72 h.

Mø have been reported to express adrenergic receptors (ADRBs; particularly ADRB2), whose signal is relayed by intracellular cAMP [40]. Therefore, we next examined the expression of ADRBs and observed that *Adrb2* was clearly expressed in propagated testicular Mø (Figure 8A). Thus, we examined β-adrenergic effects on P4 production in Mø by adding the β-adrenergic agonist Iso (1 µM) to the culture media and further measuring P4 concentrations by ELISA in the medium at 24 h and 72 h following the addition. Iso induced a significant increase in P4 concentration, 1.67 ± 0.05 (*p* < 0.001) and 1.63 ± 0.17 (*p* = 0.016) times higher than that in the untreated controls, respectively (Figure 8B); however, P4 concentration in the untreated controls at 72 h was 3.23 times higher than that at 24 h. We further examined the P4 production upregulation effect of Iso using the β2 antagonist ICI (100 nM). ICI tended to attenuate the upregulation effect, albeit there was no significant difference between the Iso and Iso plus ICI groups (*p* = 0.074) (Figure 8B). These findings indicate that β-adrenergic stimulation upregulates de novo P4 production in testicular Mø. 

### 3.8. P4 Production in Testicular Mø by M1 Polarization Inducers

We observed that testicular Mø propagated in mixed culture were highly CD206-positive and MHC II-negative, indicating characteristics of the M2 Mø phenotype [41]. Thus, we examined the effects on P4 production in propagated testicular Mø treated with the M1 polarization inducers LPS (20 ng/mL) and IFN-γ (50 ng/mL). The M1 inducers did not affect Mø densities in the culture as assessed by microscopy, although they downregulated P4 production (Figure 8C). De novo P4 production tended to decrease in the testicular Mø at 24 h following the addition of LPS plus IFN-γ, compared with that in untreated controls (0.24 ± 0.29, *p* = 0.141), and was significantly decreased at 72 h following the addition (0.13 ± 0.12, *p* = 0.039). The P4 concentration in the untreated controls at 72 h was 3.01 times higher than that at 24 h. These findings suggest that P4 production is downregulated in testicular Mø, likely polarized to the M1 phenotype. 

## 4. Discussion

### 4.1. Propagation of Testicular Interstitial Mø in Mixed Culture

We previously developed a propagation method for tissue-resident Mø for the liver, lung, spleen, and brain Mø of adult rodents [8,21]. By applying this method with modifications to the adult mouse testis, we successfully propagated testicular tissue-resident Mø by mixed culture with interstitial cells, largely composed of Leydig cells, in standard culture medium containing 10% FBS without additional growth factors. We collected >1.6 × 10^6^ testicular Mø per adult mouse, and they demonstrated high phagocytic properties and characteristic expression properties of Mø/monocyte marker membrane proteins; high expression of CD11b, CD68, CD169, and CD206; clear expression of CD115, F4/80, and Mertk; and negative expression of CD116 and MHC II. Recent studies have demonstrated that testicular interstitial Mø are CD11b-positive, CD115-positive, CD206-positive, F4/80-positive, Mertk-positive, and MHC II-negative; however, peritubular Mø are CD11b-positive, F4/80-positive, MHC II-positive, CD115-negative, CD206-negative, and Mertk-negative [11,18,23]. Thus, Mø marker expression patterns in the propagated testicular Mø were identical to those in interstitial Mø in the previous studies. Moreover, Mø classification as CD206-positive plus MHC II-negative and CD206-negative plus MHC II-positive, as used in previous studies [11,18], was standardized for identifying interstitial Mø and peritubular Mø in the testis, respectively. These classifications can also be applied to the two populations of interstitial tissue-resident Mø residing in the lung, heart, and dermis [4,42], and therefore, could represent typical phenotypes of many tissue-resident Mø at a steady state. However, CD206-negative plus MHC II-positive and CD206-positive plus MHC II-negative properties match those of the M1 and M2 phenotypes, respectively, in recruited Mø during inflammation [41]. Further studies are required to confirm this speculation regarding tissue-resident Mø.

We observed that testicular Mø propagated by mixed culture expressed not only *Csf1*, *Aldh1a2*, and *Rdh10,* but also *Ghr* and *Igf1*, which are prominent molecules involved in spermatogenesis [26,43,44,45]. These findings are supported by previous studies revealing that *Csf1*, *Aldh1a2*, and *Rdh10* are expressed in testicular Mø [45], and ALDH1A2 and RDH10, the RA synthetic enzymes, are highly expressed in interstitial Mø compared with peritubular Mø [12]. Additionally, IGF1 secreted by peritubular cells, spermatocytes, and Leydig cells promotes testosterone production in Leydig cells and proliferation/differentiation of Leydig cell progenitors [46]. Based on our newfound *Igf1* expression in propagated Mø, we hypothesized that interstitial Mø, located adjacent to Leydig cells, communicate intimately with Leydig cells via IGF1. 

Moreover, we observed that the propagated testicular Mø expressed *Tgfb1*, *Tgfbr2*, *Smad2*, and *Smad3*, suggesting that autocrine signaling via TGFβ1–TGFβR2–Smad2/3 confers interstitial Mø properties, which has already been established in several tissue-resident Mø, such as alveolar Mø, lung interstitial Mø, and microglia [25,46]. Since TGFβ1, which is produced by Sertoli, Leydig, peritubular myoid, and germ cells, regulates spermatogenesis, Leydig cell steroidogenesis, and tight junction dynamics between Sertoli cells [47], these TGFβ1 functions may also be assigned to testicular interstitial Mø. Our findings on the overall properties of marker expressions examined in the testicular Mø were completely consistent with those of testicular interstitial Mø previously reported. Thus, we believe that we can successfully propagate testicular interstitial Mø.

We examined the properties of the in vitro niche conditioned interstitial Mø in testicular interstitial cells propagated along with testicular Mø. We observed that *Csf1* and *Il34* were expressed in the mixed-cultured interstitial cells treated with the Mø depletion reagent, resulting in the negative expression of *Pu.1*. These cytokines share a common receptor, CSF1R (CD115), and promote Mø differentiation and proliferation [48,49]. We also observed CD115 and *Csf1* expression in the propagated testicular Mø, and thus, CSF1/IL34−CSF1R signaling likely promotes the propagation of testicular Mø in the mixed culture. Previous studies have revealed that CSF1 and IL34 are produced by Leydig cells [45,49]. Thus, we also determined whether Leydig cells were maintained in the mixed culture after propagation. We observed that the mixed-cultured interstitial cells treated with the Mø depletion reagent clearly expressed Leydig cell marker genes involved in steroidogenesis, except for *Cyp17a1*, *Hsd17b3*, and *Lhcgr*. These expression properties likely correspond to those of dedifferentiated adult Leydig cells and/or fetal Leydig cells: Adult Leydig cells cultured in standard culture medium lose their ability to produce testosterone within several days and produce P4 instead [50,51]. Rodent fetal Leydig cells, another population of steroidogenic cells residing in the interstitium of adult rodents, develop independently from LH and highly express *Star*, *Cyp11a1*, and *Hsd3b1*, but not *Cyp17a1* and *Hsd17b3* [46,52]. Thus, our findings on the overall properties of marker expression were largely consistent with those in adult Leydig cells and/or fetal Leydig cells; therefore, these Leydig cells were possibly mixed in the culture. Further studies are necessary to determine the features of steroidogenic cells propagated in the mixed culture. In contrast, propagated testicular Mø retained properties identical to those of interstitial Mø, suggesting that the niche suitable for testicular interstitial Mø was also maintained in the mixed culture. Thus, we believe that the niche of interstitial Mø could be successfully reproduced in vitro, and therefore, testicular-Mø-retaining properties identical to those of interstitial cells could be propagated along with steroidogenic cells mainly composed of Leydig cells. We anticipate advances in studies on male reproduction using testicular interstitial Mø in vitro in the future.

### 4.2. De Novo P4 Production by Testicular Interstitial Mø

Testicular interstitial Mø produce 25-hydroxycholesterol, which serves as a substrate for Leydig cells for testosterone production [16]. Intriguingly, we demonstrated the expression of genes involved in P4 production and that of transcription factors (SF-1 and GATA4) promoting steroidogenesis in testicular Mø. Immune cells, including Mø, in peripheral metabolic tissues, likely synthesize and metabolize steroids due to their expression of steroid synthetic/metabolic enzymes [19]. However, none of the immune cells express CYP11A1, whereas some immune cells express StAR, which triggers steroid production. Therefore, immune cells are not considered capable of producing steroids de novo. In contrast, SF-1 plays an essential role in testis development and steroidogenic enzyme production [32]: SF-1 knockdown in Leydig cells suppressed StAR and CYP11A1 expression and impaired steroidogenesis [53]. GATA4 deficiency in Leydig cells is associated with attenuated gene expression in androgen synthesis [54]. Thus, our findings suggest that testicular interstitial Mø are polymorphic cells with properties of steroidogenic cells, in which SF-1 and GATA4 possibly function as key transcription factors in steroidogenesis. Moreover, we demonstrated that testicular interstitial Mø produced P4 de novo and the other three tissue-resident Mø propagated similarly by mixed culture did not. This is the first study to demonstrate that tissue-resident Mø produce sex steroids de novo. Testicular interstitial Mø have a unique niche in interdigitation contact with Leydig cells [55,56] and are constantly exposed to a high concentration of testosterone and cytokines, which regulate steroidogenesis and spermatogenesis in the interstitial compartment [26]. Therefore, P4 production by interstitial Mø is possibly influenced by this niche. 

GJA1 is expressed in Leydig cells [37] and some tissue-resident Mø [29,30]. We detected *Gja1* expression in interstitial Mø. We also observed GJA1 immunoreactivity between CD206-positive interstitial Mø and CD206-negative cells presenting morphological properties of Leydig cells. Thus, gap junctions containing GJA1 are likely formed between Leydig cells and interstitial Mø, which may, therefore, be functionally coupled with each other via gap junction channels permeable to small molecules including metabolites and second messengers, such as cAMP [57]. We demonstrated that cAMP upregulated de novo P4 production, genes of P4 synthetic enzymes/molecules, and the transcription factors *Sf-1* and *Gata4* in testicular interstitial Mø. LH, by binding to LHCGR, promotes testosterone production in Leydig cells, where cAMP generated by the activation of adenylate cyclase via stimulatory Gs-coupled to LHCGR promotes the expression/activation of transcription factors, such as SF-1 and GATA4, to induce the expression of testosterone synthetic enzymes/molecules [39]. Thus, similar molecular machinery may operate to produce P4 in the testicular interstitial Mø when cAMP concentration is increased. Interestingly, cAMP in the interstitial Mø is supplied by Leydig cells through gap junctions; however, further studies are required to confirm this hypothesis. Moreover, we demonstrated that the testicular interstitial Mø expressed *Adrb2*, which is supported by previous studies. Immune cells are regulated by adrenergic signals and the β2-adrenergic receptor is expressed in Mø [40,58]. We also demonstrated that P4 production was upregulated by the β-adrenergic agonist Iso, and the β2-adrenergic inhibitor abrogated this upregulation to a certain extent. Thus, the β2-adrenergic system is a candidate for the regulation of P4 production in interstitial Mø.

**Figure 9:**
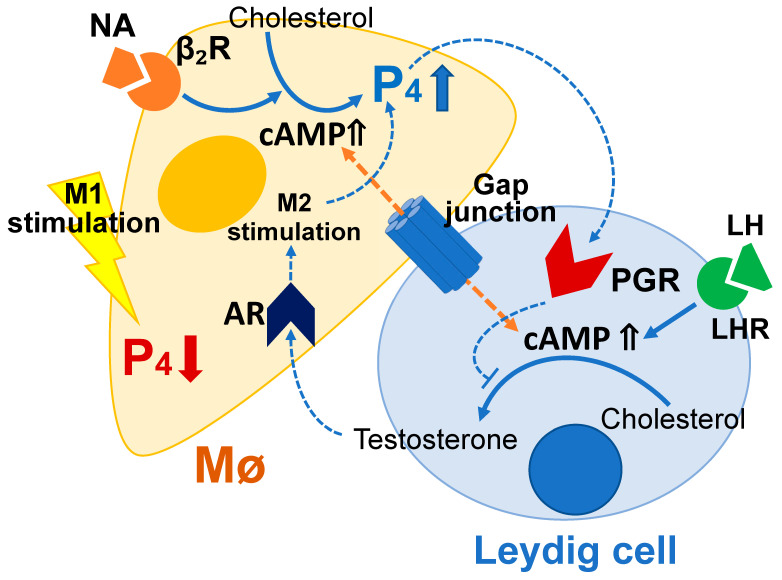
A schematic drawing showing the properties of progesterone (P4) production regulated by cyclic adenosine monophosphate (cAMP), β-adrenergic stimulation, and M1-polarization inducers in testicular interstitial macrophages (Mø). The dotted lines indicate signal cascades deduced from previous studies. The solid lines indicate well-accepted signal cascades and are derived from data in the present study.

Testicular Mø demonstrate an immunosuppressive M2 phenotype at a steady state [10]. A previous study demonstrated that alveolar Mø express AR and AR-deficient alveolar Mø demonstrated impaired M2 polarization, suggesting that androgens promote M2 polarization through AR [59]. We observed that, similar to alveolar Mø, *Ar* was expressed in the testicular interstitial Mø, which were CD206-positive and MHC II-negative, properties of the M2 phenotype in recruited Mø [41]. We also demonstrated that M1 stimulation downregulated P4 production in interstitial Mø. Based on our findings and those of previous studies, P4 production is possibly upregulated and downregulated in the M2 and M1 polarization of testicular interstitial Mø, respectively. Moreover, we demonstrated that testicular interstitial cells presenting Leydig cell features in the mixed culture expressed *Pgr*. PGR is expressed in Leydig cells [27], secondary spermatocytes, and round spermatids [60]; PGR-knockout mice exhibit increased sperm production; and PGR upregulation is associated with spermatogenesis inhibition [27]. P4 administration inhibits testosterone production in rodents, and this effect is abrogated by the P4 antagonist [61]. P4 downregulates cAMP production and gene expression driven by LHCGR in Leydig cells [62]. Based on these results, we propose that local feedback machinery between Leydig cells and adjacent interstitial Mø regulates testosterone production in Leydig cells via PGR and P4 production in the Mø via AR. Our findings, the sex steroid production controlled by interstitial Mø inferred from our findings, and evidence from previous studies are collectively summarized in Figure 9. Further studies are required to confirm our hypothesis.

## 5. Conclusions

We successfully propagated testicular tissue-resident Mø by mixed culture with interstitial cells composed of Leydig cells in standard culture medium. The testicular Mø were propagated along with interstitial cells, which exhibited properties of conditioned testicular Mø and largely corresponded to Leydig cells. Mø marker expression patterns in the propagated testicular Mø were identical to those in testicular interstitial Mø, showing properties of the M2 phenotype. Thus, the niche of testicular interstitial Mø can be successfully reproduced in vitro. Our novel findings are that (i) the interstitial Mø expressed genes involved in P4 production and transcription factors essential for steroidogenesis and produced P4 de novo, which was upregulated by cAMP and β2-adrenergic stimulation and downregulated by M1 polarization stimulation; and (ii) gap junctions were likely formed between Leydig cells and the interstitial Mø. Based on these findings, we propose that P4 production is likely upregulated and downregulated in M2 and M1 polarization of the interstitial Mø, respectively. Based on *Ar* expression in the interstitial Mø and previous evidence on PGR expression, reduced cAMP production, and gene expression via LHCGR induced by P4 in Leydig cells, we also propose that local feedback machinery between Leydig cells and adjacent interstitial Mø regulates testosterone production in Leydig cells via PGR and P4 production in the Mø via AR. Testosterone plays a leading role in the functioning of the male reproductive system, including spermatogenesis and homeostasis of reproductive organs, and exogenous P4 interferes with spermatogenesis and testosterone production. Therefore, endogenous P4 produced by tissue-resident Mø likely requires reconsideration as the control mechanism in testosterone production by central regulation of the hypothalamic-pituitary-gonadal axis [63]. This study may lead to further studies on immune-endocrine interactions in gonads related to infertility and hormonal disorders. Further in vivo and in vitro studies are required to confirm our hypothesis. 

## Data Availability

The data presented in this study are available upon request from the corresponding author.

## Supplementary Materials

The following supporting information can be downloaded at: https://www.mdpi.com/article/10.3390/biomedicines10020487/s1, Table S1, Monoclonal antibodies used for flow cytometry; Table S2, Primers of transcription factors and cycle numbers for PCR amplification; Table S3, Primers of genes involved in proliferation and characterization of macrophages and interstitial cells for PCR amplification; Table S4, Primers of genes for the characterization of macrophages for qPCR analysis.
